# Design and implementation of a community-based rehabilitation curriculum for training multidisciplinary rehabilitation teams to serve people aging with disabilities

**DOI:** 10.20407/fmj.2023-019

**Published:** 2024-10-31

**Authors:** Zaliha Omar, Yohei Otaka, Eiichi Saitoh

**Affiliations:** 1 Department of Rehabilitation Medicine, School of Medicine, Fujita Health University, Toyoake, Aichi, Japan; 2 Department of Rehabilitation Medicine, Faculty of Medicine, University Malaya, Kuala Lumpur, Malaysia

**Keywords:** Community-based rehabilitation, Curriculum design, Community engagement, Learner, Rehabilitation team

## Abstract

**Objectives:**

We aimed to design and implement a community-based rehabilitation (CBR) curriculum to promote community engagement by multidisciplinary teams.

**Methods:**

Participants in this prospective interventional study at a rehabilitation institution for people aging with disabilities included learners, the chief executive officer of the institution, program auditors, and community members. A customized CBR curriculum was developed using systems thinking design. Thirty-five learners were trained through 36 instructional contact hours and 60 hours of guided self-directed learning. Learners completed pre-training self-reported questionnaires regarding knowledge and experience of CBR. During training, learners were evaluated continuously through observation, assignments, self-reported feedback questionnaires, and CBR projects. The chief executive officer was interviewed during the study. The program auditors were interviewed and wrote reports on the curriculum and observations regarding the CBR projects. Learners reported on community participation in these projects.

**Results:**

Thirty-three of 35 learners completed the program, 31 (94%) of whom had no prior knowledge of CBR. Learners implemented nine community engagement CBR projects, in which 1,293 community members participated. The auditors commended the curriculum content and its positive impact on learners and the community. The chief executive officer implemented inclusive community engagement at work. A CBR curriculum was dynamically developed for multidisciplinary rehabilitation team training to promote community engagement.

**Conclusions:**

The custom-designed CBR curriculum enabled multidisciplinary teams to practice community engagement at work. Equipped with CBR knowledge and skills, teams engaged with multiple sectors of the community to enhance patients’ rehabilitation potential and increase public awareness through the implemented projects.

## Introduction

There are 1 billion persons with disabilities (PWD) worldwide,^1^ many of whom have benefited from community-based rehabilitation (CBR).^2,3^ The concept of CBR has been widely adopted worldwide to address the rehabilitation needs of PWD through decentralized resources and community participation. CBR has evolved from a strategy for the implementation of the Convention of the Rights of PWD to an approach for inclusive community development.^4^ In short, CBR is person-centered care based in the community that requires a multimodal approach and collaborative effort.^5^

In Japan, the increasing population of older people has further increased the number of people aging with disabilities.^6^ Conventionally, CBR is practiced as a community effort in which people from a community are brought together and empowered to create an activity that benefits PWD in the community. However, this is not the way community outreach for rehabilitation services typically functions in Japan. Rather, when individuals in communities in Japan require rehabilitation, such services are typically delivered from their respective bases (e.g., rehabilitation hospitals), including in communities in remote areas. Advancements in service provision and technology in rehabilitation institutions have enabled the delivery of clinical multidisciplinary care. Japan contained 186,504 physical therapists in 2023,^7^ 108,872 occupational therapists in 2023 (estimate),^8^ 1,734,000 nurses in 2020,^9^ and 3,963 physiatrists (Board-certified and Board-certified members of Rehabilitation Medicine) in 2022.^10^ Rehabilitation practitioners generally do not receive formal training in community engagement, which can be defined as “the process of working collaboratively with groups of people who are affiliated by geographic proximity, special interest and similar situations with respect to issues affecting their wellbeing”.^11^

It has been estimated that 2.4 billion people worldwide could currently benefit from rehabilitation.^12^ Japan introduced a community-based integrated care system in 2012,^13^ and its revision in 2017 emphasized building a cohesive society.^14^ Community engagement is one approach for promoting active community participation in rehabilitation. The concepts, principles, and practice of CBR bring together diverse sectors that can enhance a culture of community engagement. A past study reported that inclusion of a CBR curriculum in Bachelor’s honors degree programs in occupational therapy and special education resulted in meaningful activities for PWD in the community.^15^ CBR training of community workers and volunteers worldwide has provided PWD with beneficial skills,^16–19^ encouraging developed countries to use the CBR approach for geriatric rehabilitation.^20,21^ In the context of person-centered care using multimodal intervention and applying a collaborative approach in rehabilitation, community engagement skills are essential, but are rarely acquired from formal education. To the best of our knowledge, no previous studies have reported community engagement curricula in structured undergraduate rehabilitation studies.

The authors considered that CBR training for the multidisciplinary staff of a rehabilitation institution may be useful for promoting a community engagement culture. The operational definition of CBR in this study was community engagement. Therefore, this study aimed to design and implement a CBR curriculum that can provide multidisciplinary rehabilitation teams with skills to engage with the community they serve. The CBR curriculum was designed and implemented as postgraduate education for multidisciplinary rehabilitation personnel. The scarcity of literature on the subject led us to implement a qualitative method at the outset to avoid missing the wealth and depth of information in its naturistic form.^22^

## Methods

### Ethical considerations

Ethical approval of the protocol was obtained from the institution (10th October, 2019; Ethics Committee/Internal Review Board of Amano Rehabilitation Hospital, Hatsukaichi, Hiroshima; approval application number: 0004).

Written consent was obtained from the chief executive officer (CEO), the program auditors, learners, and community members. The study was conducted in accordance with the principles embodied in the Declaration of Helsinki.

### Study design and setting

This mixed-methods prospective interventional study was conducted between August 2019 and March 2022 at a rehabilitation institution in Hiroshima, Japan that provides medical rehabilitation services for people aging with disabilities. The rehabilitation institution is a single corporation that provides a spectrum of rehabilitative services through a rehabilitation hospital, rehabilitation clinic, day-care center, residential care center, and community rehabilitation service center. Although the role of each service center remains distinct, there is a continuum of flow of patients, staff and work processes from one center to another, and a seamless workflow between the centers. The institution serves as a single entity, and the CBR curriculum was custom-designed to meet the main goal of the institution as a provider of rehabilitation services that seeks to promote community engagement through its mission to work alongside the community, as stipulated in its goals. A total of 588 multidisciplinary staff worked at these centers.

### Participants

Participants in this study comprised learners, the CEO of the institution, three program auditors, and community members. Community members included all persons aging with disabilities (i.e., all age groups of people who were aging with disabilities), in addition to older persons. The inclusion criteria were: 1) willingness to participate in the study, 2) either sex, and 3) all age groups. Exclusion criteria were 1) inability to participate fully for any reason, and 2) inability to complete participation in the study. A convenience sample of 35 learners was selected from staff by management to undergo the CBR curriculum. These learners had been identified as key implementers of the community based integrated care system for the institution, and were divided into three cohorts. Three program auditors were appointed by the CEO, all of whom had skills and experience in community engagement. A senior physical therapist with CBR experience audited the first cohort, an academic nurse educator audited the second cohort, and a community physical therapist audited the third cohort. Community members comprised clients from the institution, their families, and the neighborhood population.

The authors played no role in the selection of participants.

### Development of CBR curriculum content

#### Framework

The curriculum was designed using a systems thinking design approach,^23^ Tyler’s principles of learning,^24,25^ and lessons from Sawyer’s emerging learning sciences.^26^ Rehabilitation of people aging with disabilities calls for person-centered care and awareness of the diverse range of services they require. Such services may function through specific systems and frameworks.^27^ It is therefore helpful to understand the concept of systems functioning during community engagement activities. Skills that can enhance handling of complex systems are embedded in systems thinking design. Hence, the curriculum was designed using a systems thinking design approach^23^ encompassing hard, soft, and critical thinking. Hard systems that were incorporated into the curriculum included frameworks that have been established specifically in rehabilitation practice, including the United Nations International Year of Disabled Persons, United Nations Convention on the Rights of PWD, United Nations Sustainable Development Goals (SDGs), United Nations Economic and Social Commission for the Asia Pacific decades for PWD, Millennium Development Goals, Incheon Strategy, International Classification of Functioning Disability and Health, Rehabilitation 2030, and the World Health Organization Active Aging Framework. Soft systems thinking takes a stepwise method of problem-solving, including identification and description of “problems,” development of key definitions, creation of conceptual models and comparison with real situations, assessing feasibility, as well as defining changes and implementation of proposed changes. Critical thinking systems involve systematic processing of problem situations by exploring a variety of perspectives and identifying primary and secondary issues, and intervention strategies that follow a consideration of systems approaches, methodologies, models, and structures. Finally, intervention and monitoring of progress is implemented by evaluating improvements, reflecting on systems approaches, and discussing and agreeing on the next steps.

The process of learning can be made effective by applying well-grounded and established concepts and principles, such as Tyler’s principles of learning.^24,25^ Developing a curriculum as problem elements, providing experience, and approaching assessment as evaluation, then delivering the objectives and contents as activities that are organized, and culminating in evaluation, can make learning meaningful to learners. Learning science is key to efficiency in acquisition of knowledge and skills. Learning can be offered through conceptual and procedural knowledge, reinforming of lessons, immediate feedback, story-telling approaches, learners’ involvement, and measurement of results. Emerging technologies do not make a difference in overall outcomes of learning if the sciences are not adhered to.^26^

Further examples of systems thinking were provided by reflecting on the 40-year personal CBR experience of the first author working in Malaysia and internationally. This experience includes advocacy work on 1) empowerment of PWD through a contemporary CBR project involving an information network for PWD,^28,29^ 2) alternative and augmentative communication for inclusive literacy, 3) inclusive sports (e.g., Wheelchair Tennis Malaysia),^30^ 4) a therapeutic sensory stimulation garden,^31,32^ and 5) accessibility. All learners were intended to receive 36 contact hours of class lessons over 1 year, consisting of one 3-hour class per month. Cohort 1 (10 learners) underwent training as planned. However, because of coronavirus disease 2019 (COVID-19) pandemic-related restrictions, the schedule was changed for Cohort 2 (12 learners) and Cohort 3 (13 learners). Although Cohort 2 took 18 months and Cohort 3 took 13 months to complete their training, the contact hours of class lessons with the sole instructor (the first author) and guided self-directed learning for all cohorts remained the same. Four classes for Cohort 3 were completed virtually.

#### Components and elements

Components of the curriculum including the objectives, content, evaluation, and respective elements are described in Table 1. The detailed elements for the curriculum content, including didactic topics and assignments, are shown in Table 2. The corresponding expected behavior change of learners is shown in a two-dimensional chart, on the basis of Tyler’s method.^24,25^ Learners’ behavior changes can be expected from knowledge and experience gained from specific curriculum contents. These changes range from an understanding of concepts and principles to the ability to apply them to real situations and pay attention to the social needs of clients. The learning modalities used in the current study included learner-centered active participation, community instruction, as well as problem-based and project-based learning. A sample of the program schedule for the 12 3-hour class sessions is shown in Supplementary Table 1.

#### Learners’ feedback

At the end of each class, learners completed a feedback questionnaire. Responses were discussed in the subsequent class. The topics that were incorporated into the curriculum following those responses included: 1) volunteerism, 2) sustainability, 3) return on investment, and 4) cultural aspects of communication. Twelve feedback questionnaires were prepared for each cohort, and a sample feedback questionnaire is shown in Supplementary Table 2.

#### Evaluation of learners

Continuous evaluation of learners was performed on the basis of their presentations, self-reported responses to feedback questionnaires, class assignments, and project-based assignments. At the end of training, each learner wrote a report on their learning experience. The report format included the headings noted in Supplementary Table 1 (session 9). Learners also submitted CBR project team reports, the format of which is shown in Supplementary Table 1 (session 7). Results of the learners’ assignments, feedback responses, and reports were quantitatively analyzed, and learners’ comments were recorded as qualitative statements.

#### Institutional leadership

The CEO was interviewed before the start of the training to ascertain the institution’s mission, which was incorporated into the curriculum. Semi-structured follow-up interviews were held with the CEO during the study. Open-ended questions allowed the CEO to bring up new ideas. The first author conducted the interviews with the CEO and recorded the responses.

#### Community participation

In the planning of the CBR projects, learners were instructed to incorporate participation of the community through needs assessment, shared decisions, and the implementation of projects. Local community needs were assessed through surveys and questionnaires designed by the learners. The findings were the focus of the CBR projects. For two of the projects on community awareness of long-term care insurance, the teams based their needs assessment on the results of a published online survey, which was performed by Nippon Life Insurance Company via a website from October 1–27, 2013 (10,129 respondents, 56% female). Community members participated as speakers, participants, or leaders of group activities in each project.

#### Medium of instruction

Training sessions were conducted in English. Three professionals translated all of the training materials, including lecture notes, audio-visual slides, questionnaires, and verbal communication from English to Japanese for learners. All content prepared by learners in Japanese was then translated into English for the instructor.

#### Outcome measures

The outcomes of the implementation of the CBR curriculum were quantitatively and qualitatively analyzed. Quantitative outcome measures included: 1) the proportion of learners having acquired community engagement knowledge and skillsets before and after training, 2) the number of CBR projects, and 3) the number of community members who participated in the projects. Qualitative outcome measures included participants’ records of interviews and feedback responses.

#### Data analysis

Qualitative analysis was conducted on the following collected data: 1) records for development of the CBR curriculum, 2) participants’ interviews and narratives, 3) learners’ self-reported feedback questionnaires, assignments, and reports, and 4) observations of community members’ participation. Quantitative data were input into an Excel spreadsheet (Microsoft Corporation, Redmond, WA, USA) and analyzed.

## Results

Learners’ characteristics are shown in Table 3. At the end of the study, 33 of 35 learners completed the CBR training by March 2022. One learner dropped off because of illness and another became pregnant and had related health issues. Of the remaining 33 learners, 31 (94%) had no prior knowledge of CBR. Of these learners, only two had worked in CBR in the past; both had attended related conferences, and one had collaborated in the early stages of various CBR projects. Analysis of the self-reported feedback questionnaires that were completed by learners at the end of each contact session (total of 12 for each participant) showed that all 33 learners who completed the study had progressively acquired knowledge, skills, attitudes, and confidence in performing community engagement activities at work. Nine CBR projects were implemented; their outcomes and effects on the community are shown in Table 4. All learners reported that they will continue to perform community engagement at work. The results of the interviews with the CEO of the institution indicated that there was management support for sustaining CBR culture at the institution, as shown in Table 5.

The needs of the local community, as described by the learners in the questionnaires we administered, included: 1) a detailed understanding of long-term care insurance and the disability pension, 2) practical aspects of nutrition and swallowing, 3) socialization opportunities for older persons and the superaged, 4) accessible shopping ideas for affordable daily living items, 5) sources for universal design items frequently used by people aging with disabilities, and 6) maintaining fitness at home. Examples of comments made by community members who participated in the nine projects are illustrated in Table 6. Thematic analysis of these comments revealed three main categories: functional capability, namely self-empowerment (e.g., projects 3 and 6), increased public awareness of issues commonly experienced by PWD and their families (e.g., project 1), and joy and happiness as value-added outcomes in rehabilitation (e.g., projects 4 and 5).

The auditor for Cohort 1 commented, “This is a novel program. Our therapists usually do not have a community engagement skillset, so this type of CBR training can fill the gap in community participation for establishing community-based integrated care systems. The curriculum is well-designed, original, and appropriate for an institution that is implementing such a system. I believe the curriculum design is an effective way to enhance the knowledge of physical therapists and occupational therapists who often work in silos. The lack of concern for practical community issues faced by PWD can be solved through a custom-designed CBR curriculum, as has been done in this study.” The auditor for Cohort 2 remarked, “It is important to be conscious of community needs in rehabilitation. We must be sensitive about the need to communicate effectively when dealing with the community and during interactions, such as in public seminars. The communication component of the CBR curriculum can enhance this soft skill, which is much needed in community engagement.” The auditor for Cohort 3 commented, “Hearing real people’s voices is key to inclusive community development. It is good that you have done this in today’s seminar.”

## Discussion

The purpose of the current study was to design and implement a CBR curriculum for equipping multidisciplinary team members at a rehabilitation institution with community engagement skills to meet the institution’s mission of being community oriented when serving its clients. To the best of our knowledge, this is the first study of CBR to examine the training of multidisciplinary teams at work. Our findings confirmed that the curriculum, which was developed from a generic concept of CBR and rehabilitation medicine to an authentic customized curriculum by utilizing input from participants using a systems thinking design approach, can culminate in CBR projects through community engagement. Remarks made by community members and auditors highlighted the empowering capacity and capability of community engagement by learners. The thematic analysis (self-empowerment, public awareness, and value-added rehabilitation outcomes of joy and happiness) indicated positive outcomes and effects on the community through CBR training of multidisciplinary rehabilitation team members.

When designing a curriculum, its self-sufficiency, significance, learnability, utility, validity, and consistencies with social realities should be considered.^24^ Overall, the curriculum content of this study satisfies all considerations, and was improved over the 3-year period of curriculum development. The principles of learning experience included variety, interest, relevance to life, suitability, and comprehensiveness. Cumulative development behavior that learners gradually experience during an educational process often arises from organized curriculum content and learning experiences.^24,25^ In the current study, although all learners received the same curriculum content and total hours of learning, the time period of study for each cohort was different because of restrictions related to the COVID-19 pandemic. Despite a major difference in organization of the course, the vertical and horizontal organizational principles remained the same. The course also adhered to the criteria of continuity, sequence, and integration. Hence, all learners met the main learning objectives of acquisition of knowledge, skills, and attitude to be engaged in the community at work.

Past studies have reported positive effects of CBR training for volunteers and workers in the community.^33,34^ In contrast, in the current study, learners of the CBR curriculum were multidisciplinary rehabilitation teams at work in an institution, and the effect of community engagement was felt in the community. Therefore, the CBR curriculum, which was designed to meet the needs of implementers of community engagement activities at work, was useful for learners, the institution, and the community at large. It adopted a behavioral change approach^24,25^ in learners and nurtured them into the community engagement culture at work. Lessons from studies on CBR training pedagogic approaches^35^ and cultural considerations^36^ were incorporated into the curriculum design, which also adopted a systems thinking design approach.^23^ The soft systems thinking approach was helpful for adapting the delivery of teaching to meet the demands of a diverse range of age, experience, communication, culture, and spiritual beliefs between and among learners, translators, and the instructor. The hard systems thinking approach, using established protocols, frameworks, and standards, was believed to be necessary for future development of evidence-based practice of CBR at work.

Previous studies on CBR curriculums for physical therapy and occupational therapy students, and CBR workers in the community have been performed, but none have been as detailed and clearly described as the current study. Project-based learning has previously been reported to be successful.^15^ In Fiji and the Solomon Islands, a CBR curriculum model was developed for community workers and was delivered through an intensive 5-day workshop.^35^ This model was reported to be effective for empowering participants to develop community-inclusive projects for PWD.^35^ Similarities between this previous study and the current study include the following: 1) the content was based on the concepts of CBR, communication, and culture; 2) active participatory learning, problem-based learning, and project-based learning, and 3) empowerment capacity imparted to learners.

Past studies of CBR have not discussed plans for sustainability of the culture of community engagement.^15–17^ The content of our curriculum included elements of sustainability and social return on investment. Interviews with the CEO revealed definitive plans for sustaining community engagement activities and the culture of CBR at the institution. Examples included: 1) a plan to create a CBR division at the institution, 2) inclusion of CBR activities in the credentialling process by the Commission on Accreditation of Rehabilitation Facilities, and 3) enhancing the community-based integrated care system at the institution before 2025 by leveraging the community engagement skillset that has been acquired by learners. As in any systems design method, a systems thinking design approach to developing a curriculum should emphasize sustainability.^23^ The need for health systems to include rehabilitation services across the care continuum and patients’ lifespans is crucial, especially considering that 2.4 billion people worldwide could benefit from these services.^37^ Evidence for the benefits of rehabilitation and related factors, like community engagement at work, suggests the need to incorporate rehabilitation into welfare, health, and education policies and practice.

It is uncommon for CBR or community instruction modules to be included in undergraduate curriculums. In Malaysia, community instruction has been provided since the inception of the first medical school at the University Malaya in 1964 through the social and preventive medicine curriculum.^38^ In 1984, a rehabilitation medicine component enhanced community instruction through the introduction of CBR, and academic capacity-building ensured that it was sustained.^39^ For medical students in the United States, community-based experience with PWD was introduced in 2008, and this was the beginning of community engagement experience for many students^40^. In Japan, community-related curricula have been described in medical schools^41^ and public health nursing studies^42^ later, after the introduction of the community-based integrated care system in 2012.^43^ Strong leadership is crucial for a culture of CBR and community engagement to be continually developed. This need has been suggested by the vanguard rural town of Miyagi, Japan, since the 1970s.^13^

In the current study, learners were selected by the institution to meet the goal of implementing a community-based integrated care system. No previous studies identified in the literature described the selection process of learners in CBR training. Future studies should investigate enrollment statistics and make comparisons between professions and cultural backgrounds.

One implication of the current study findings is that due consideration should be given to non-clinical aspects of patients’ lives. Additionally, drawing on multisectoral community collaboration can enhance their rehabilitation potential. The current study partially reflects the importance of naturistic settings for a complex representation of real-world rehabilitation practice.

One strength of the current study lies in the pragmatic and dynamic considerations of the curriculum design in meeting the needs of the institution, learners, and community members. The main limitation of the curriculum was that communication between learners and the instructor was indirect, through a team of three translators, potentially affecting the accuracy and precision of certain terms. Additionally, cultural differences between a Malaysian instructor and Japanese learners, community members and translators could potentially lead to misinterpretation. Furthermore, the selection of learners and the appointment of auditors by top management could potentially cause biases in the questionnaire responses and reports, as respondents may be hesitant to make negative comments about the program. Other challenges included the restrictions related to the COVID-19 pandemic, a lack of funding, language barriers, and cultural differences. These challenges were balanced by the maturity of learners, administrative support from top management, eagerness of members of the community to share their thoughts, and the vast experience of the translators. Self-reported outcomes of acquisition of knowledge, skills and attitudes of learners revealed that gradual progress was achieved over the course of 12 sessions of the CBR curriculum. This learning provided a basic foundation for learners’ community engagement skills, which can be built up over time in the future, and is embedded in the philosophy of the institution: “Service for community with the community.” The curriculum could be further improved by reducing communication barriers, implementing delivery by selected qualified staff from the organization and internationally, and using more robust quantitative markers for outcomes of knowledge, skills, and attitudes of learners. While this CBR curriculum may not be suitable for universal use, we believe it could be adapted to other communities by modifying its design to tailor it to the specific needs of the place, learners, and local community.

In conclusion, the current findings showed the feasibility of designing and using a customized CBR curriculum to train multidisciplinary rehabilitation teams to practice community engagement at work. On the basis of these results, we hope that the identification and interpretation of observations can make way for robust quantitative studies in the future. Randomized controlled trials are warranted to further assess the effects of the CBR curriculum on learners, the institution, and the community. Future studies should be conducted to monitor the sustainability of the program, and to assess the return on investment of regular training. Additionally, CBR curriculum design studies on similar groups of multi-professional rehabilitation teams in other settings may be useful because results can then be compared, standards set, and evidence-based practices initiated. The usefulness of community engagement activities for the implementation of the community-based integrated care system in Japan remains to be seen.

## Figures and Tables

**Table1:** Community-based rehabilitation curriculum for learners

Component	Description
Objectives	• To equip learners with knowledge, skills, and attitudes to practice community-engagement at work.
	• To enable learners to create community engagement activities on the basis of person-centered needs.
Learning outcomes for sessions 1–12	The curriculum stepped up cumulatively to meet the objectives described above, helping learners to instill interest, become immersed, acquire formative knowledge, deeply understand the concept of community-based rehabilitation, learn the principles for applying this concept to situational needs, and apply these principles in formulating community engagement activities at work, culminating in community-based rehabilitation projects during project-based learning.
Content	Selected topics and subjects that principles of learning, learning sciences, and systems thinking approach were applied to.
Pre-session reading topics	Learners had to access pre-session reading topics from the Internet. These were given a week before classes.
	Examples
	• United Nations Convention on the Rights of Persons with Disabilities
	• Japan Disability Act
	• United Nations Economic and Social Commission for Asia Pacific decades of Persons with Disabilities
	• Community-based rehabilitation matrix
	• International Classification of Functioning, Disability, and Health
	• United Nations Sustainable Development Goals
Didactic	Classes and Zoom lecture materials were translated into Japanese and distributed to learners 3–7 days before the scheduled lectures.
Group work	Interactive, reflective, and problem-based learning were created through groups and teamwork.
	• Learners were paired and discussed topics for 10 min, then gave a 2-min presentation to the class. Topics included volunteerism, care for older persons, and intergenerational culture.
	• Groups of 3–4 learners were given topics related to the didactic topic for the day; after a 15–20-min discussion, a 5-min summary was presented to the class, e.g., long-term care insurance and daily living items.
	• Teams chose projects related to community-based rehabilitation and organized and implemented them.
Evaluation	
Individual learners’ assignments	• Observations of the learners’ participation in class were recorded.
	• Feedback questionnaires were completed at the end of each class, and responses were incorporated into the curriculum.
	• Assignments (e.g., concept of community-based rehabilitation and principles of rehabilitation) were submitted to the instructor before the next class.
	• The learner provided comments on other learners’ presentations.
	• The analyses were shared with all learners.
Teams’ assignments	• Cohorts 1, 2, and 3 consisted of 2, 3, and 4 teams, respectively.
	• Teams chose community-based rehabilitation projects and applied the knowledge gained during the training to organize and implement them.

**Table2:** Two-dimensional chart of didactic topics and assignments in the curriculum content, and the expected behavioral change of learners

Curriculum content		Expected behavioral change							
		Understand concept	Understand principles	Ability to apply to a real situation	Ability to discuss	Feel confident understanding	Attention to social needs	Familiar with reliable resources	Ability to report results
A	Didactic topic								
1	CBR: a. Concept, b. Matrix	X	X	X	X	X	X	X	X
2	Communication	X	X	X	X	X	X		X
3	Community	X	X	X	X	X	X	X	X
4	Community engagement	X	X	X	X	X	X	X	X
5	Culture	X	X	X	X	X	X		X
6	Evolution of CBR	X		X	X	X	X	X	X
7	ICF	X	X	X	X	X	X	X	X
8	Inclusive community development	X	X	X	X	X	X	X	X
9	Rehabilitation competency framework	X	X	X	X	X	X	X	X
10	Rehabilitation: PCC, multi-modal, collaborative	X	X	X	X	X	X	X	X
11	Rehabilitation 2030	X	X	X	X	X	X	X	X
12	Social return on investment	X	X	X	X	X	X		X
13	Sustainability-UNSDG	X	X	X	X	X	X	X	X
14	UNESCAP decades of PWD	X	X	X	X	X	X	X	X
15	Universal design	X	X	X	X	X	X	X	X
16	Volunteerism	X	X	X	X	X	X	X	X
B	Assignment								
1	Individual								
	a. Pre-session reading				X			X	X
	b. Assignments 1–5	X	X	X		X		X	X
2	Group work								
	a. Paired and group deliberation, presentation, and critique				X		X	X	X
	b. Team projects	X	X	X	X	X	X	X	X

CBR, community-based rehabilitation ICF, International Classification of Function, Disability and Health; PCC, person-centered care; UNSDG, United Nations Sustainable Development Goals; UNESCAP, United Nations Economic and Social Commission of Asia Pacific; PWD, persons with disabilities.

**Table3:** Learners’ characteristics (n=35)

Characteristic	Value
Sex	
Male, n (%)	11 (31.4%)
Female, n (%)	24 (68.6%)
Duration worked at the institution, mean (range)	12 years, 4 months (1 year, 5 months to 27 years, 8 months)
Profession	
Physician	1 (2.9%)
Nurse	8 (22.9%)
Physical therapist	10 (28.6%)
Occupational therapist	2 (5.7%)
Speech-language-hearing therapist	2 (5.7%)
Social worker	2 (5.7%)
Clinical psychologist	1 (2.9%)
Care worker	2 (5.7%)
Administrator	7 (20.0%)
Prior knowledge of community-based rehabilitation	2 (5.7%) (one physician and one occupational therapist)

**Table4:** Community-based rehabilitation projects conducted by learners

No.	Cohort team	Contents of the project	Number of community participants (n=1,293)	Effect on the community^a^
1	Cohort 1 Team 1	Public awareness campaign on long-term care insurance (part 1)	132	Two educational videos have been uploaded to the institution’s website and YouTube. The videos will be developed into a series that can benefit people of all ages. Cohort 3 Team 3 expanded this campaign.
2	Cohort 1 Team 2	A workshop on disability pension for childhood disability Participants included parents of children with disabilities and the lay public	120	Parents were given “Pink files” to record events from birth to age 20 years. In this way, cumulative records of information required for application of the pension is readily available for use when the child reaches 20 years of age.
3	Cohort 2 Team 1	Swallowing rehabilitation seminar: a public awareness and education campaign for people with swallowing problems	76	Planned for an annual event in the future, to be available to people with swallowing concerns from any cause, their caregivers, and the lay public.
4	Cohort 2 Team 2	A wellness information station and an online version of the health, fitness, and rehabilitation education program for people of all ages	672	COVID-19 pandemic restrictions pending, this initiative was designed to revert to a yearly autumn festival activity focused on inclusive community educational programs on health, fitness, and rehabilitation.
5	Cohort 2 Team 3	Dementia Café aimed at preparing and promoting socialization among people with dementia and their caregivers	83	This is a regular feature for a transition activity from inpatient rehabilitation to community-based rehabilitation. It will continue to provide support activities and socialization opportunities for patients after discharge from inpatient rehabilitation.
6	Cohort 3 Team 1	A seminar: graduates of high school–what next? The focus was on teenagers with disabilities seeking employment	36	A follow-up project focused on an economic empowerment program for high school graduates among youths with disabilities was planned.
7	Cohort 3 Team 2	Advocacy for regular intergenerational exchange activities at a day-care rehabilitation center	54	A booklet on plans for intergenerational exchange activities three times per year was prepared and will be implemented.
8	Cohort 3 Team 3	Public awareness campaign focused on long-term care insurance (part 2)	91	A series of videos on long-term care insurance for increasing public awareness is being planned.
9	Cohort 3 Team 4	Advocacy on volunteerism for a therapeutic sensory stimulation garden project at a nursing home	29	Two manuals on activities in the garden that have been prepared will be used by volunteers and staff at the nursing home.

^a^ Activities that continue to be performed or have potential ongoing benefits for the community. COVID-19, coronavirus disease 2019; no., number.

**Table5:** Interviews with the chief executive officer

Timing of the interview	Question	Answer	Quotation
At the start of the study (August 2019)	Please share the vision, mission, and goals of your institution.	“Number one is to be community oriented when we serve our clients.”	“I hope CBR training can enable our staff to support us to do [the] CBICS before 2025.”
Completion of training of Cohort 1 (October 2020)	Please comment on the outcome of the training at this stage.	“I am very happy to learn that CBR-trained staff now understand CBR.”	“I am so happy to see [that] CBR-trained staff are implementing [the] CBICS with confidence.”
Six months after completion of training of Cohort 1 (April 2021)	Do you think CBR training is in line with your vision, mission, and goals?	“Yes, most definitely. Our hospital board and I are so pleased.”	“I will make sure that staff continue the CBR projects and engage with our local community, so that our clients have a [sic] peace of mind.”
Twelve months after completion of training of Cohort 1 (October 2021)	Would you like to share future plans for CBR at your organization?	“CBR will be a regular activity at our organization. It will be included in the Commission for Accreditation of Rehabilitation Facilities report as [a] continuing improvement program.”	“I want to create a new CBR division so that CBR can continue to grow, and especially help young people aging with disabilities.”
		“I am so thrilled that we will organize the 2023 National Japan CBR & Care Conference in Hiroshima, especially because all of our CBR-trained staff will help us prepare for it.”	“Moving forward, we want to work on branding our city as inclusive and universally designed through CBR and community engagement. We will collaborate with city hall and our community to make it a reality.”

CBR, community-based rehabilitation; CBICS, community-based integrated care system.

**Table6:** Examples of comments obtained by community members who participated in the CBR projects

Project no.	Question/Situation	Answer/Comments
1 (Long-term care insurance video)	A 36-year-old man was asked what he thought of the video.	“The cartoon video is short and easy to watch. It alerted me about long-term care insurance. My parents may need it when they require care one day, especially as we live longer these days. I look forward to learning more about it, also for myself in the future.”
1 (Long-term care insurance video)	A 15-year-old student watched the long-term care insurance video with her grandmother. She was asked to comment.	“It was very easy to understand. The short story makes me want to learn more about long-term care insurance. I want to help my grandparents and my parents if they need them [sic]. I hope there will be more stories to follow.”
1 (Long-term care insurance video)	A 75-year-old woman was asked to comment on the video.	“I think many Japanese [people] depend too much on the government to take care of them. This video makes me think we also have a responsibility to take care of ourselves as we grow old. I want to learn more.”
2 (Workshop on disability pension)	A mother of a child with a congenital abnormality was asked what she liked best about the workshop.	“I didn’t know that it was necessary to keep detailed records of my baby’s progress for many years as she grows because I [sic] need them when I fill the forms for the disability pension application when she is 20 years old. Many friends have told me that the application procedure is very complex. I am grateful and relieved that I get [sic] this information at this workshop. I shall share this information with other mothers. I will discuss [it] with other mothers if I have any uncertainties.”
3 (Seminar on swallowing)	A participant was invited to make a comment.	“I learnt new information about nutrition and swallowing problems. I must share this information with others. I also know who I can get help from when I get [sic] some trouble with food and swallowing.”
4 (Wellness information station)	A 67-year-old gentleman remarked excitedly.	“This quiz is fun. I now know that I do not have enough knowledge about aging health, so I must try to learn more from others who know. I will discuss these questions when I meet my friends next. I wonder if they know more than I do.”
5 (Hidamari café at a hospital)	A patient was focused on the activity, filling a pouch with dried lavender and smiling. She was heard making a voluntary remark.	“I love the smell of this lavender. This is so very nice! I want to keep doing more!”
6 (Seminar on employment for teenagers with disabilities)	A mother spoke about her experience bringing up her teenage son with a disability. Her son remarked spontaneously.	“Next time, I want to talk about myself.”

CBR, community-based rehabilitation; no., number. Note: Interviews were conducted by the first author in English, or through a translator. Responses were recorded by the first author.
