# Supplementary material for: Design and implementation of a community-based rehabilitation curriculum for training multidisciplinary rehabilitation teams to serve people aging with disabilities
Source: Fujita Med J. 2024 Oct 31;11(1):1–10. doi: 10.20407/fmj.2023-019 (PMC11782937; doi:10.20407/fmj.2023-019)
Supplement: Supplementary file 1 — Supplementary Tables [file fmj-11-001-s001.pdf]

Supplementary Table 1. Program schedules for 12 successive 3-hour class sessions（Cohort 3)

Session 1: Introduction to CBR (Part 1)		
0900–0910	Opening remarks	Learning outcomes:<br>1. To generate interest in CBR<br>2. To understand CBR in depth<br>3. To be able to define CBR as stated in the Joint Statement by the WHO, ILO and UNESCO<br>4. Being staff of the institution, realizing the importance of understanding CBR.
0910–1010	Breaking the ice session.	Aim: to warm learners up to the training program. Everyone, including the instructor, was given 2 minutes to introduce themselves. They shared their profession, place, duration and nature of work, thoughts on CBR and English proficiency.
	Discussion on pre-session reading	Emphasis was placed on making time for self-directed learning. Pre-session reading materials were discussed. Learners spoke about their understanding of the concept of CBR, United Nations Convention of the Rights of PWD, Japan Disability Act., and CBR in Japan.
1010–1020	Break	
1020–1050	Didactic 1 (Part 1)	Early beginnings of CBR projects that their institution had embarked on or collaborated with nationally and internationally. Aim: to instill a sense of pride regarding top management initiating CBR practice, to motivate participation.
1050–1105	Group work: Deliberation 1	Group A comprised learners who had worked at the institution for more than 5 years. Group B comprised learners who had worked at the institution for less than 5 years.<br>Task: for learners to identify a community engagement activity they had personally been involved in. The activity could be related or unrelated to their job. Deliberations were within and between groups.
1105–1125	Presentation on group work	One person from each group presented a summary of their deliberations. Other learners critiqued the presentations and shared their views with the class.
1125–1155	Didactic 1 (Part 2)	CBR explained. Dictionary definition, concepts, principles, and practice. Real-world experiences of the instructor were used as examples.
1155–1200	Assignment 1<br>Feedback 1	To reflect on the concept of CBR; to prepare for assignment 2 after session 2.<br>Learners completed and submitted self-reported feedback questionnaire 1.

Session 2: Introduction to CBR (Part 2)		
0900–0910	Opening remarks	Reflections from session 1: Instructor observations and feedback 1, which had been analyzed, were discussed.<br>Learning outcomes:<br>1. To understand the term CBR<br>2. To be able to define CBR<br>3. To know that CBR evolves over time<br>4. To be able to identify milestones in the evolution of CBR worldwide<br>5. To feel motivated to do self-readings on evolution of CBR internationally
0910–1010	Deliberation 2: Discussion on pre-session reading.	On the basis of the analyses of feedback 1, the pre-session reading instructions remained the same as those for session 1. Four volunteers summarized what they had read and shared their thoughts on the subjects. Other learners shared critiques regarding the presentations.
1010–1020	Break	
1020–1100	Didactic 2: Introduction to CBR	Reflections on the early beginnings of community-based rehabilitation initiated by the chief executive officer of the institution from 2017 to 2019. The therapeutic sensory stimulation garden at the institution's nursing home was highlighted as an example of a CBR project. The concepts, principles, and practice of CBR and rehabilitation were explained in depth using these examples.

1100–1145	Group work	Experience-sharing regarding past community engagement activities and preparation for presentation; one person per group presented their discussions.
1145–1200	Assignment 2<br>Feedback 2	Question: In your own words, please describe what you understand by the term CBR. Please refer to the 2004 joint position definition of CBR by the WHO, ILO and UNESCO.<br>Learners completed and submitted self-reported feedback questionnaire 2.

Session 3: Evolution of CBR: international perspective		
0900–0910	Opening remarks	Reflect on feedback 2 on the basis of quantitative and qualitative analyses.<br>Learning outcomes:<br>1. To be able to recall and appreciate milestones in the evolution of CBR worldwide<br>2. To understand the significance of the Biwako Millennium Framework of action<br>3. To appreciate the need for the Incheon Strategy<br>4. To understand the importance of the United Nations Millennium Developmental Goals<br>5. To be motivated to read about the above
0910–0930	Comments and discussions on pre-session reading materials and Assignment 2	Reading materials:<br>1. United Nations International Year of Disabled Persons 1981<br>2. United UNESCAP<br>3. United Nations World Program of Action Concerning Disabled Persons 1982<br>4. United Nations Standard Rules on the Equalization of Opportunities for Persons with Disabilities (Standard Rules UN 1993)<br>5. Biwako Millennium Framework of Action<br>6. Incheon Strategy<br>7. Millennium Development Goals.<br>Answers submitted by learners on assignment 2 implied that they had read and understood the first two pre-session reading materials and met the learning outcomes of session 2.
0930–1030	Didactic 3 (Part 1)	CBR at the institution in relation to the evolution of CBR internationally. UNESCAP and the four decades of PWD in the Asia Pacific region were introduced. The continuum from clinical hospital-based rehabilitation principles and practice to CBR was explained.
1030–1045	Break	
1045–1145	Didactic 3 (Part 2).	The concept of CBR Matrix was explained. This concept was then applied to work at the institution that continued into community extended services for clients aging with disabilities. Concepts and principles of universal design and their application to CBR were explained, and examples were given.
1145–1200	Explanation of Assignment 3	Questions:<br>1. What do you know about the LTCI system in Japan?<br>2. Have you ever heard anyone discussing LTCI? If yes, what was the discussion about?<br>Learners were divided into three groups to work on Assignment 3: to focus on ways to increase awareness of long-term care insurance in the community for people of all ages (0 to 100).
	Feedback 3	Learners completed and submitted self-reported feedback questionnaire 3.

Session 4: Making major systems inclusive of persons with disabilities through CBR		
0900–0910	Opening remarks	Reflections on sessions 1, 2, and 3: Explanation on a buildup of learning process on CBR using established international hard systems as developed through UNESCAP. Emphasis on soft and critical systems thinking approach through learners' active participation.<br>Learning outcomes:<br>1. To be able to think through the evolution of CBR internationally<br>2. To understand CBR evolutionary milestones in relation to diverse circumstances worldwide

		3. To be able to recall examples of community-based inclusive development models using CBR approaches outside of Japan<br>4. To understand the CBR Matrix<br>5. To appreciate the significance of the United Nations SDG in inclusive community development<br>6. To be mindfully prepared for the CBR project
0910–0940	Feedback response	Discussion of feedback 3: The instructor commended the positive feedback on learners' ability to understand the concept of CBR and their confidence in applying it at work. Learners expressed appreciation for the guided team decisions on the four CBR projects that will be undertaken as community engagement activities for project-based learning.
	Comments on pre-session reading materials session 2 and 3	Learners began to realize that the pre-session reading materials had served as a prelude to the didactic and deliberation sessions, making the latter two much more comprehensible. Comments made by learners concurred with the instructor's thoughts regarding the aptness of using the frameworks through the UNESCAP decades for PWD in illustrating the evolution of CBR towards inclusive community development. Moving forward, merging the needs of PWD into SDG made this issue clearer from an inclusive perspective.
	Comments on Assignment 3	Assignment 3 focused on Japan's LTCI. Learners' presentations implied that their knowledge of LTCI was limited. Some learners assumed that clients who needed to know about LTCI could use resources in social welfare departments and through the care management systems. However, they were unsure whether this assumption was true for their patients.
0940–1030	Didactic 4 (Part 1)	Making major systems inclusive of PWD through CBR. Learners were guided into hard systems thinking that could be applied in UNESCAP decades 1–4, including the Biwako Millennium Framework of Action, Incheon Strategy. and Millennium Development Goals.
1030–1040	Break	
1040–1050	Didactic 4 (Part 2).	This session expanded on Didactic 4 Part 1 to focus on inclusive development frameworks (e.g., CBR matrix, person-centered care rehabilitation approach, and principles of universal design). The sky mirror model of presentation was used, citing the instructor's personal experiences of inclusive development in Malaysia and internationally.
1050–1145	Introduction to Assignment 4<br>Prepare for team presentations	Project-based learning commenced with team assignments as follows:<br>Team 1: "Information dissemination seminar for people with disabilities" continued the seminar series initiated by Cohort 1 Team 2. The team chose a topic regarding meeting the needs of high school leavers with disabilities.<br>Team 2: "Intergenerational exchange activities" between children of a nursery school and older people and PWD who use a neighboring day care facility.<br>Team 3: "Long term care insurance picture book and video" was used as a succession plan for the Cohort 1 Team 1 CBR project. They collaborated with the Cohort 1 team leader.<br>Team 4: "TSSG," which aimed to develop a sustainable plan for a project that had been set up in 2019 at a nursing home.
1145–1155	Presentation 4	One representative from each team presented their first deliberations on the respective projects described above. Comments were made by the instructor and provided back to the learners.
1155–1200	Feedback 4	Learners completed and submitted self-reported feedback questionnaire 4.

Session 5: Systems approach for inclusive development for persons with disability through CBR		
0900–0910	Opening remarks	Learning outcomes:<br>1. Understanding of CBR Matrix<br>2. Ability to reflect on the significance of the United Nations SDGs in inclusive community development<br>3. Consideration of community-based integrated care system in terms of CBR and inclusive community development
0910–0930	Feedback responses from session 4	Quantitative analysis of feedback from session 4 showed that learners had acquired adequate CBR knowledge. Qualitative analysis of comments by individual learners further confirmed this conclusion. Additionally, the

		images of real people used as examples in the didactic sessions made the exercise more emotionally effective.
0930–1020	Didactic 5: Systems approach for inclusive community development of PWD through CBR	In the last four sessions, the instructor cited target actions in the Incheon Strategy, Biwako Millennium Framework, and Millennium Development Goals as part of evolution of CBR. These turned out to be frameworks exclusively for PWD that helped prepare them for inclusive development in the United Nations SDGs.
1020–1030	Break	
1030–1130	Deliberation and preparation of CBR team project	Learners gathered in their respective teams and worked on their CBR projects.
1130–1155	Presentation 5	Each team's representative presented the progress made in the team project. The instructor and all learners provided comments.
1155–1200	Feedback 5	Learners completed and submitted self-reported feedback questionnaire 5.
**Session 6: Preliminary plans for project-based learning**		
0900–0910	Opening remarks	The goal for the day was to make observable progress in the respective team projects.
0910–0925	Feedback response from session 5	Quantitative analysis showed learners' deeper understanding of the concept of CBR through project-based learning. Learners could apply lessons learnt to their team projects. Two themes from qualitative analysis were that the intra- and inter-group discussions were "stimulating" and "motivating."
0925–1030	Group work<br>Preparation for presentation on preliminary team work	Teams continued working on their CBR projects. The instructor was at hand to guide and help as needed. Practical questions were posed and guidance was given. Solutions remained the responsibility of team members.
1030–1040	Break	
1040–1120	Presentation on CBR projects by teams 1 and 3	Team 1 was progressing well with the information sharing project about high school leavers with disabilities seeking jobs. Teams found instructor and peer comments constructive and motivating.<br>Team 3 shared their progress on an LTCI picture book and video. One issue raised was the difficulty for team members to meet outside of the dedicated training hours because of work commitments. This difficulty was partially resolved by learners working together online most of the time, and in-person during the CBR training class.
1120–1150	Presentation on CBR projects for teams 2 and 4	Team 2 updated the class on "intergenerational exchange activities." The nursery children had made preparations to act as postal workers who will deliver cards to older people at a day care facility.<br>Team 4 presented their plans on promotion of the TSSG in a nursing home of their institution. Suggestions to sustain volunteerism had to be shelved because of COVID-19 pandemic restrictions. Hence, the team planned to develop manuals on the maintenance of the garden. The instructor and non-presenting learners provided comments on the presentations.
1155–1200	Feedback 6	Learners completed and submitted self-reported feedback questionnaire 6.
**Session 7: Planning CBR projects by four teams, and preparation for team report writing**		
0900–0910	Opening remarks	The goal was to reflect on achievements to date, to motivate learners, and for learners to realize their plans for four CBR projects by the end of the training.
0910–0940	Reflection on session 6 and pause to think about achievements to date and make future plans for the project-based learning	Because the learners were halfway through the CBR training, it was an appropriate time to reflect on activities to date, and future activities. Learners had shown ability to apply the knowledge and skills gained by conceptualizing four CBR projects, which had to be completed at the end of their training.
0940–1030	Didactic 7:<br>The headings to be included in the team report:<br>1. Background<br>2. Introduction<br>3. Team CBR Project<br>4. Impact	Team reports were to be submitted after the team projects had been launched. The focus was on collaborative effort in realizing community engagement activities through CBR projects.<br>• Description of backdrop of the CBR training in relation to the mission, vision, and goals of the institution by referring to the institution website.<br>• Description of the training program. The concept of CBR, as learned

	5. Sustenance<br>6. Continuing improvement plans<br>7. Discussion<br>8. Conclusion<br>9. Acknowledgment<br>10. References<br>Appendix	already in the course, and the challenges and limitations faced throughout the course.<br>• The project title and process of working as a team on the CBR project.<br>• Reason for choosing that specific project and steps taken to realize it. Steps taken to launch the project.<br>• Outcome: Number of people who benefitted directly and indirectly from the project. Means of measuring outcomes.<br>• Plans by the team to follow-up on the project after the launch.<br>• Impact on the community using CBR matrix. Potential use of new skills to implement community-based integrated care system. Beneficiaries and means of measurement.<br>• Impact on the learner, the team and the institution. Return on investment: tangible and social/intangible returns to their institution, to Japan as a country, and to the world as a whole.<br>• To think along the lines of Rehabilitation 2030 and the United Nations SDGs.<br>• Others: The team's succession plans. Challenges and limitations faced throughout the implementation of the team project.<br>• To list all sources of information linked to the project that had been used.
1030–1040	Break	
1040–1140	Team work	Teams continued working on their respective CBR projects. The instructor was at hand to guide and help when needed.
1120–1155	Presentation on progress of CBR projects for teams 1, 2, 3 and 4	A representative from each team presented updates on the progress of their team projects. The instructor provided comments and peer group feedback was provided by non-presenting teams.
1155–1200	Feedback 7	Learners completed and submitted self-reported feedback questionnaire 7.

Session 8: Updating plans for community-based rehabilitation projects		
0900–0910	Opening remarks	Goal for the day: to establish progress for the CBR team projects.
0910–0940	Reflection on session 7	All learners continued to show progress in applying the theory learnt to their respective team projects. In response to the question "has the team clearly explained about their project," peers provided constructive comments. When asked to comment on the needs assessment components of the presenting teams' project work, most learners felt that they were appropriate and adequate.
0940–1030	Team work	As in session 7.
1030–1040	Break	
1040–1110	Rehearsal by team 3 for launch.	A video of an example of an intergenerational activity held at day care for older persons was shown. Peer-group critiques were helpful. Contingency plans had been made for the possibility of COVID-19 pandemic-related restrictions. The team responded well to questions by peers and the instructor. The team appeared to be ready for the launch as planned.
1110–1150	Presentation on CBR projects by all teams<br><br>Comments by instructor.	Team 1: Seminar, "High school graduation: then and now" was ready to be held. A hybrid format was planned. Speakers included a welfare officer from the community, mothers, and a high school graduate.<br>Team 2: Plans for the intergenerational exchange activity were presented. Older participants taught children games that are usually played on New Year's Day, in which children sang and danced.<br>Team 3: Feedback regarding the YouTube video on LTCI was provided by staff of the institution. The original plan was to get feedback from the institution's clients and the community but this was not possible because of COVID-19 restrictions. Plans for promotion to a wider population will be implemented in future.<br>Team 4: Details of the TSSG manuals were presented; one for maintenance and another for cultivation. Elements of the promotion of volunteerism were incorporated.<br>All teams had applied community engagement principles in their CBR projects.

1150–1200	Feedback 8	Learners completed and submitted self-reported feedback questionnaire 8.
Session 9: Plans for implementation of team projects, and individual training report		
0900–0910	Opening remarks	Goals:<br>• To continue showing progress in team projects<br>• To prepare for individual learner reports
0910–0940	Comments on feedback 8 (Peer-group / instructor)	Self-reported feedback indicated that participation of all team members in the respective CBR projects was good, and positive progress was made. Peers learnt a lot from listening to the presentations of others. Learners reported that the instructor’s comments were simple and easy to understand.
0940–1030	Team work	Continuation of team project work.
1030–1040	Break	
1040–1110	Didactic 8:<br>Format of individual report writing with headings:<br>1. Background<br>2. Introduction<br>3. Progress through the training<br>4. Challenges and limitations<br>5. Impact<br>6. Sustenance<br>7. Conclusion<br>8. Acknowledgment	• Description of selection as learners, roles, pre-training preparation.<br>• Description of the main professional training program and additional training, and any training programs prior to joining the institution.<br>• Description of any training programs attended since working. Prior exposure to CBR, and thoughts about CBR before entering the program and throughout the training.<br>• Experience working as a team member of the CBR team project.<br>• Challenges and limitations faced throughout the course.<br>• Compliance with instructions. Contribution as a member of a team project. New lessons learnt through the CBR training. Description of possible application of concept of CBR at present job.<br>• Any communication or cultural issues between learners and the instructor.<br>• Community engagement knowledge and skills as a professional at work.<br>• Sustaining and continual improvement of CBR culture at work.
1110–1150	Presentation on CBR projects	All teams continued to progress steadily in their projects.
1150–1200	Feedback 9	Learners completed and submitted self-reported feedback questionnaire 9.
Session 10: Progress check for implementation of team CBR projects		
0900–0910	Opening remarks	The goal for the day was to consolidate all plans that had been made and prepare the launch of team projects.
0910–0940	Comments on feedback 9 (Peer-group / instructor)	Session 9, which had been conducted entirely via a virtual platform on the part of the instructor was well received by learners. Interactions were positive, and having the translators in the same room as the learners was helpful.
0940–1030	Sharing the experience of CBR project team 1	Team 1 presented the launch of their seminar and shared their experience with the class. The other three teams presented on the progress of their respective CBR projects.
1030–1040	Break	
1040–1150	Team work	Continuation of CBR project team work of teams 2, 3, and 4 to prepare for launch. Team 1 started their team project report writing.
1150–1200	Feedback 10	Learners completed and submitted self-reported feedback questionnaire 10.
Session 11: Plans for launching of remaining team projects		
0900–0910	Opening remarks	The instructor commended the observable competitiveness in the performance of the four teams.
0910–0930	Comments on feedback 10 (Peer-group / instructor)	Quantitative analysis showed good progress made in project-based learning despite restrictions related to the COVID-19 pandemic. Qualitative analyses indicated positive learning experiences and adaptability of learners in implementing the community engagement activities.
0930–1015	Restaging seminar by team 1 for cohort 3 learners	Because of work and time constraints, when team 1 conducted their seminar, only the instructor and an external auditor were present. Hence, a video of the seminar was shared with the whole class so others could learn from it.

1015–1025	Break	
1025–1125	Team work	On the basis of feedback regarding time limitations outside the class sessions, this section was created so that teams could work together on unfinished projects.
1125–1155	Presentation on CBR projects	Teams 2, 3, and 4 gave updates and progress of their CBR projects. The instructor and peers made constructive comments.
1155–1200	Feedback 11	Learners completed and submitted self-reported feedback questionnaire 11.

Session 12: Final preparations for launching the remaining team projects and conclusion		
0900–0905	Opening remarks	The instructor commended learners for their perseverance and hard work towards completion of the CBR training.
0905–0915	Comments on feedback 11	Detailed analyses were shared with learners. These analyses were positive with respect to critiques and conduct of project-based learning.
0915–1030	Team work	Team 3 performed final preparation for presentation to the class. The other teams worked on their respective team reports for the CBR training.
1030–1040	Break	
1040–1110	Concluding didactic	The training program was summarized so that learners could have a holistic view of the CBR training that they had undergone. Learners had acquired knowledge and skills to embark on community engagement activities at work, and the evaluation confirmed that they had developed the appropriate attitudes to practice these skills. Learners were challenged to take responsibility for undertaking this work, as Japan is a world leader in rehabilitation. They were advised to think of universal design in the broadest sense of the word and inclusive development in the course of their work. Areas in which learners could contribute to their institution included social return on investment, incorporation of CBR work into Commission for the Accreditation of Rehabilitation Facilities, preparation for CBR and Care Conference 2023, and preparation for a community-based integrated care system before 2025.
1110–1140	Continue team work<br>Prepare for presentation	Team 3 continued preparation for their presentation to the class. Other teams continued working on their respective team reports of the CBR training, which were to be submitted within a month of completion of the training.
1140–1155	Presentation on CBR project	Team 3 presented their storyline for a video (version 3) on public awareness of long-term care insurance and a booklet on a similar topic. Comments were provided by the instructor and peer group feedback was provided by non-presenting teams.
1155–1230	Feedback 12	Learners completed and submitted self-reported feedback questionnaire 12. This concluding feedback questionnaire was more comprehensive than those for the preceding sessions.

CBR, Community-based rehabilitation; WHO, World Health Organization; ILO, International Labor Organization; UNESCO, United Nations Educational, Educational, Scientific and Cultural Organization; PWD, persons with disabilities; UNESCAP, United Nations Economic and Social Commission for Asia Pacific; LTCI, long-term care insurance; SDG, Sustainable Development Goals; TSSG, Therapeutic sensory stimulation garden; COVID-19, coronavirus disease 2019.

Supplementary Table 2. An example of a feedback questionnaire at the end of a class for learners – Cohort 3 Session 12, March 18, 2022

Name:
Please answer all questions.

A. Please mark the appropriate answer corresponding to each choice.

		Strongly agree	Agree	Unsure	Disagree	Strongly disagree
1	Overall, are the goals set at the beginning of the course met?	☐	☐	☐	☐	☐
2	Overall, are the learning objectives met?	☐	☐	☐	☐	☐
3	Overall, do you find the format (e.g., pre-session reading, didactic/group deliberation, presentation) of each session stimulating?	☐	☐	☐	☐	☐
4	Overall, do you think the style of the delivery of the course is appropriate?	☐	☐	☐	☐	☐
5	Overall, are the didactic sessions clear?	☐	☐	☐	☐	☐
6	Overall, are the didactic sessions useful for your work?	☐	☐	☐	☐	☐
7	Did you discover new things about your co-participants that you did not know before?	☐	☐	☐	☐	☐
8	Regarding the timing of the course delivery, is the fortnightly session suitable?	☐	☐	☐	☐	☐
9	Is a 3-hour session adequate to absorb the course content?	☐	☐	☐	☐	☐
10	Did you learn new things that you did not previously know about rehabilitation in the course?	☐	☐	☐	☐	☐
11	Will you be able to use this knowledge to improve your work further?	☐	☐	☐	☐	☐
12	Can the course contribute to improvement of your work?	☐	☐	☐	☐	☐
13	Will you be able to use CBR concepts in your work?	☐	☐	☐	☐	☐
14	Will you participate in work related to CBR for your institution in the future?	☐	☐	☐	☐	☐
15	Did the training cause any disruption in your current work?	☐	☐	☐	☐	☐
16	Did you get good value for the time you spent away from your regular work?	☐	☐	☐	☐	☐
17	Do you think the training is appropriate for staff at your institution?	☐	☐	☐	☐	☐
18	Do you think the training can help your institution further improve its ability to serve the community?	☐	☐	☐	☐	☐
19	Do you think your institution gets value for money in running this training?	☐	☐	☐	☐	☐
20	Do you think the community that your institution serves can benefit from CBR?	☐	☐	☐	☐	☐

B. Please answer the following:

1. Did the course change your past impression of CBR? ☐Yes☐No

Did it make your impression more positive? ☐Yes☐No

Did it make your impression less positive? ☐Yes☐No

2. How much time do you think goes into preparation of each session for the consultant trainer?

☐Less than 10 hours
☐10–20 hours
☐20–30 hours
☐30–40 hours
☐More than 40 hours

3. Did the COVID-19 pandemic cause any problems in the running of the CBR training course?

For you personally ☐Yes☐No

For your institution ☐Yes☐No

4. What is your overall opinion of the 12-month CBR training program?

☐Excellent
☐Very good
☐Good
☐Fair
☐Bad

C. Please write a page or less, in your own words, about the 12-month CBR training.

a. Overall comments b. Good points c. Weaknesses d. Any other matters e. Comments on Zoom session

Thank you very much for your responses.
